# Pattern of cancer risk in persons with AIDS in Italy in the HAART era

**DOI:** 10.1038/sj.bjc.6604923

**Published:** 2009-02-17

**Authors:** L Dal Maso, J Polesel, D Serraino, M Lise, P Piselli, F Falcini, A Russo, T Intrieri, M Vercelli, P Zambon, G Tagliabue, R Zanetti, M Federico, R M Limina, L Mangone, V De Lisi, F Stracci, S Ferretti, S Piffer, M Budroni, A Donato, A Giacomin, F Bellù, M Fusco, A Madeddu, S Vitarelli, R Tessandori, R Tumino, B Suligoi, S Franceschi

**Affiliations:** 1 Epidemiology and Biostatistics Unit, Centro di Riferimento Oncologico IRCCS, Via Gallini 2, Aviano 33081, Italy; 2 Friuli Venezia Giulia Cancer Registry, Agenzia Regionale di Sanità, Via Pozzuolo 330, Udine 33100, Italy; 3 Epidemiology Department, INMI ‘L Spallanzani’ IRCCS, Via Portuense 292, Rome 00149, Italy; 4 Romagna Cancer Registry, Department of Medical Oncology, Romagna Cancer Institute (IRST), Via Piero Maroncelli, 34/36, Meldola 47014, Italy; 5 Cancer Registry of Milan, Epidemiology Unit, Local Health Autority of Milan, Corso Italia 19, Milan 20122, Italy; 6 Tuscany Cancer Registry, Unit of Epidemiology, Research Institute of the Tuscany Region, Via di S Salvi 12, Florence 50135, Italy; 7 Genoa Province Cancer Registry and Genova University, Istituto Nazionale per la Ricerca sul Cancro IRCCS, Largo Rosanna Benzi 10, Genoa 16132, Italy; 8 Università di Padova, Registro Tumori del Veneto, Istituto Oncologico Veneto IRCCS, Passaggio Gaudenzio 1, Padua 35131, Italy; 9 Registro Tumori Lombardia – Provincia di Varese, Istituto Nazionale Tumori, Via Venezian 1, Milan 20133, Italy; 10 Piedmont Cancer Registry, City of Torino, Centro di Prevenzione Oncologica, Via San Francesco da Paola 31, Torino 10123, Italy; 11 Modena Cancer Registry, Department of Oncology and Haematology, University of Modena and Reggio Emilia, Via Dal Pozzo 71, Modena 41100, Italy; 12 Brescia Health Unit Cancer Registry, Via Cantore 20, Brescia 25128, Italy; 13 Reggio Emilia Cancer Registry, Department of Public Health, Via Amendola 2, Reggio Emilia 42100, Italy; 14 Parma Province Cancer Registry, Azienda Ospedaliera di Parma, Via dell’Abbeveratoia 4, Parma 43100, Italy; 15 Umbria Cancer Registry, Dept. of Surg. Med. and Public Health, University of Perugia, Via del Giochetto, Perugia 06100, Italy; 16 Ferrara Cancer Registry, Sez. Anatomia Patologica, Dip. Med. Sperimentale & Diagnostica, Ferrara University, Via Fossato di Mortara 64, Ferrara 44100, Italy; 17 Trento Cancer Registry, Osservatorio Epidemiologico, Viale Verona, Trento 38100, Italy; 18 Cancer Registry of Sassari (ASL1), Via Tempio 5, Sassari 07100, Italy; 19 Salerno Cancer Registry, via Loria 24, Salerno 84129, Italy; 20 Piedmont Cancer Registry, Province of Biella, Epidemiology Unit – Prevention Department ASL 12, Via Don Sturzo 20, Biella 13900, Italy; 21 Alto Adige/Südtirol Cancer Registry, Corso Italia 13/M, Bolzano 39100, Italy; 22 Campania Cancer Registry, Azienda Sanitaria Locale Napoli 4, Piazza San Giovanni, Brusciano (NA) 80031, Italy; 23 Syracuse Province Registry of Pathology (RTP), Corso Gelone 17, Syracuse 96100, Italy; 24 Macerata Province Cancer and Mortality Registry, Dip. Medicina Sperimentale e Sanità Pubblica, Camerino University, Via Gentile III da Fabriano, Camerino (MC) 62032, Italy; 25 Sondrio Cancer Registry, Azienda Sanitaria Locale, Via Sauro 38, Sondrio 23100, Italy; 26 Ragusa Cancer Registry, Department of Oncology – Azienda Ospedaliera ‘Civile M.P.Arezzo’, Via Dante 109, Ragusa 97100, Italy; 27 National Institurte of Health, Viale Regina Elena 299, Rome 00161, Italy; 28 International Agency for Research on Cancer, 150 cours Albert Thomas, Lyon cedex 08 69372, France

**Keywords:** AIDS, epidemiology, HAART, human papillomavirus, hepatitis viruses

## Abstract

A record-linkage study was carried out between the Italian AIDS Registry and 24 Italian cancer registries to compare cancer excess among persons with HIV/AIDS (PWHA) before and after the introduction of highly active antiretroviral therapy (HAART) in 1996. Standardised incidence ratios (SIR) were computed in 21951 AIDS cases aged 16–69 years reported between 1986 and 2005. Of 101 669 person-years available, 45 026 were after 1996. SIR for Kaposi sarcoma (KS) and non-Hodgkin lymphoma greatly decreased in 1997–2004 compared with 1986–1996, but high SIRs for KS persisted in the increasingly large fraction of PWHA who had an interval of <1 year between first HIV-positive test and AIDS diagnosis. A significant excess of liver cancer (SIR=6.4) emerged in 1997–2004, whereas the SIRs for cancer of the cervix (41.5), anus (44.0), lung (4.1), brain (3.2), skin (non-melanoma, 1.8), Hodgkin lymphoma (20.7), myeloma (3.9), and non-AIDS-defining cancers (2.2) were similarly elevated in the two periods. The excess of some potentially preventable cancers in PWHA suggests that HAART use must be accompanied by cancer-prevention strategies, notably antismoking and cervical cancer screening programmes. Improvements in the timely identification of HIV-positive individuals are also a priority in Italy to avoid the adverse consequences of delayed HAART use.

Three types of cancer that occur in HIV-positive individuals, namely Kaposi sarcoma (KS), non-Hodgkin lymphoma (NHL), and invasive cervical cancer (ICC), are currently included in the European clinical AIDS definition (Ancelle-Park, 1993). However, excesses of some non-AIDS defining cancers have been consistently reported in persons with HIV/AIDS (PWHA), in particular Hodgkin lymphoma (HL), and cancers of the anus, lung, and liver (Grulich *et al*, 2007).

After the introduction of the highly active antiretroviral therapy (HAART) in 1996, huge declines in KS and NHL incidence have been consistently reported in high-resource countries (Grulich *et al*, 2002; Franceschi *et al*, 2003, 2008; Engels *et al*, 2008; Polesel *et al*, 2008). The ultimate influence of the partial immune reconstitution and improved survival made possible by HAART on the risk of ICC and non-AIDS-defining cancers, notably HL, anal and liver cancer is, however, still unclear (Herida *et al*, 2003; Clifford *et al*, 2005; Dal Maso *et al*, 2005; Biggar *et al*, 2006; Engels *et al*, 2006; Hessol *et al*, 2007; Patel *et al*, 2008).

In Italy, a high-quality centralised AIDS Registry is active on a nationwide scale (Centro Operativo AIDS, 2008), whereas cancer registries (CRs) cover nearly one-third of the population (Curado *et al*, 2007). The aim of the present study was to provide updated information on cancer excess in Italian PWHA after the introduction of HAART, and compare it with corresponding findings prior to 1997. Attention will also be paid to the cancer pattern among the growing proportion of late presenters; that is, PWHA whose first HIV-positive test was concomitant with AIDS diagnosis (>50% of new AIDS cases since 2002 in Italy, Centro Operativo AIDS, 2008).

## Materials and methods

The general design of our record-linkage study has been described previously (Franceschi *et al*, 1998; Dal Maso *et al*, 2003). In brief, reporting of AIDS cases to the Italian AIDS Registry started in 1982 on a voluntary basis and became mandatory in November 1986. At the end of 2005, a total of 57 531 AIDS cases had been reported nationwide (Centro Operativo AIDS, 2008). The AIDS Registry has been recording information on CD4+ cell count, and HAART use at AIDS diagnosis, since 1990 and 1999, respectively, and that on first HIV-positive test since 1996.

A network of CRs has been active in Italy since the early 1980s (AIRT Working Group, 2006). In the late 1990s, 24 CRs had been established and included a population of 17.3 million (30% of the total Italian population, Table 1, Curado *et al*, 2007). Cancer registries vary both in size, covering populations of approximately 180 000 to nearly 2.1 million, and in duration of activity (Table 1). Routine indicators of data completeness and quality in Italian CRs are, however, satisfactory (Curado *et al*, 2007).

Record linkage between the AIDS Registry and CRs was performed using an updated version of an ‘*ad hoc*’ software application designed previously and validated (Dal Maso *et al*, 2001). Briefly, records were linked by last and first name, and by date of birth. The name–date algorithm required: (a) that the records be identical for at least one critical field and (b) that the other two critical fields, if not identical, differ only in prescribed ways. The procedures removed all personal identifiers and, hence, registry staff was blinded to which persons had been linked.

The present study was restricted to AIDS patients who: (1) were diagnosed with AIDS between 1986 and 2005; (2) were aged between 16 and 69 years at the time of AIDS diagnosis and (3) reported a ‘legal residence’ in areas covered by a CR. Person-years at risk were computed between 5 years prior to AIDS diagnosis, and the date of cancer or death or 10 years after AIDS diagnosis, whichever occurred earlier. This interval was left or right censored if no complete CR data were available for the corresponding years. To reduce losses to follow-up, dates of death were updated through record linkage with the National Mortality Database.

Observed cases included incident cancer cases reported to CRs during the above-defined person-years at risk. Cancer site and type were classified according to the International Classification of Disease, 10th revision (World Health Organisation, 1992) and were checked for quality by CR coordinators. The basis of diagnosis was reported either as microscopic confirmation, including histological, haematological, or cytological confirmation, or as other, that is, clinical, instrumental diagnosis, or death-certificate-only. When an AIDS-defining cancer was mentioned in both the AIDS Registry and a CR, the earliest date of cancer diagnosis was retained. When KS was reported in a CR before the date of AIDS diagnosis in the AIDS Registry, AIDS onset was backdated. The same was done for NHL and ICC when they had been reported to a CR within 5 and 2 years, respectively, before AIDS diagnosis.

Expected numbers of different cancers were computed for each CR from sex-, age-, and period-specific incidence rates (Parkin *et al*, 1992, 1997, 2002; Curado *et al*, 2007). Observed numbers of cancer in PWHA were compared with expected numbers by means of standardised incidence ratios (SIRs), and corresponding 95% confidence intervals (CI) were computed using the Poisson distribution (Breslow and Day, 1987).

SIR were calculated for calendar period, distinguishing the pre-HAART (1986–1996) from the post-HAART (1997–2004) period. For 1997–2004, and for cancers showing a significantly increased risk and at least 10 cases, SIRs were also computed separately by age group (16–34, and 35–69 years), HIV transmission category (injecting drug users (IDUs), men who have sex with men (MSM), heterosexuals) and time of cancer occurrence in respect to AIDS diagnosis (4–60 months before, from 3 months before to 3 months after, 4–60 months after, and 61–120 months after). SIR were also computed separately by interval between first HIV-positive test and AIDS diagnosis (<1, 1–9, ⩾10 years), and country of birth (Italy or other).

## Results

A total of 21 951 AIDS cases (78% men and 22% women) were reported in Italy between 1986 and 2005 in areas covered by a CR (Table 1). The number of person-years available (56 643 and 45 026, respectively), as well as number of cancers reported (1098 and 897), was similar in 1986–1996 and 1997–2004 (Table 2). However, the proportion of IDUs (63 and 42%, respectively) and the median age (32 and 38 years) varied substantially in the two periods, as did the relative importance of different cancer types. Kaposi sarcoma and NHL represented 84.4% of all cancers in 1986–1996, but 72.0% in 1997–2004. Marked declines in SIR emerged for KS (from 1792 to 572, respectively) and NHL (from 497 to 93), whereas the SIR for the combination of non-AIDS-defining cancers did not change (2.4; 95% CI: 2.0–2.8 and 2.2; 95% CI: 1.9–2.5).

A significantly elevated risk emerged in 1997–2004 for cancer of the liver (6.4; 95% CI: 3.7–10.5) and penis (12.0; 95% CI: 2.3–35.5), whereas the excess risk for leukaemia disappeared. Elevated SIRs for cancer of the anus (44.0; 95% CI: 21.8–78.9), vulva and vagina (24.3; 95% CI: 4.6–71.8), lung (4.1; 95% CI: 2.9–5.5), brain (3.2; 95% CI: 1.4–6.3), skin (non-melanoma, 1.8; 95% CI:1.2–2.6), HL (20.7; 95% CI: 14.6–28.5), and multiple myeloma (3.9; 95% CI: 1.0–10.0) in 1997–2004 were similar to those found in 1986–1996 (Table 2). The comparison between the two periods was not modified by the exclusion of CRs that contributed information for the most recent period only (data not shown).

Persons with HIV/AIDS born outside Italy contributed 15% of person-years and 7.8% of cancer cases in 1997–2004. They showed similar SIR for AIDS-defining illnesses and slightly lower SIR of non-AIDS-defining cancers (1.5; 95% CI: 0.7–2.4) than PWHA born in Italy (data not shown).

Microscopic confirmation was available after 1996 for all ICC, anal cancer, and HL (16 mixed cellularity, 7 nodular sclerosis, and 14 HL of unspecified type), as well as 79% of lung cancer. Eleven out of 16 liver cancers were microscopically or instrumentally confirmed. Microscopic confirmation was available for only one (a glioma) out of eight brain tumours, and seven had a concomitant AIDS-defining illness in the brain (six toxoplasmosis and one leukoencephalopathy).

**Figure 1:**
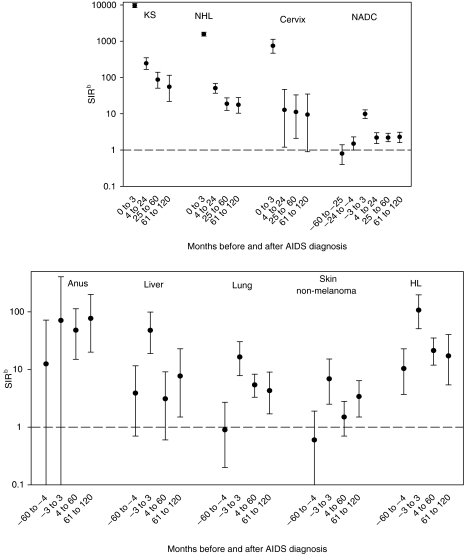
Standardised incidence ratio (SIR) and corresponding 95% confidence interval of selected cancers in persons with HIV/AIDS by time of cancer occurrence with respect to AIDS diagnosis. Italy, 1997–2004^a^. Abbreviations: KS: Kaposi sarcoma, NHL: non-Hodgkin lymphoma, NADC: non-AIDS-defining cancers, HL: Hodgkin lymphoma. ^a^Cancers reported to cancer registries in people with AIDS, aged 16–69 years, from 5 years prior to 10 years after AIDS diagnosis (at/after AIDS for AIDS-defining cancers). ^b^Vertical bars represent 95% confidence intervals.

For both AIDS- and non-AIDS-defining cancers the highest SIR emerged in the 3 months prior to or after AIDS diagnosis (Figure 1). Prior to AIDS diagnosis, a significant risk excess was only seen for HL (SIR=11.2; 95% CI: 4.5–23.3), whereas elevated SIRs emerged for all examined cancers 4–120 months after AIDS diagnosis.

SIR years for KS, NHL, and HL were lower among PWHA younger than 35 compared with older ones, whereas those for non-AIDS-defining cancers other than HL were higher (Table 3). Women showed higher SIR of KS, NHL, and cancer of the liver and lung than men, whereas the opposite was found for HL. With respect to HIV transmission category, SIRs were especially high for cancer of the liver and lung among IDUs, and for KS and HL among MSM. For all non-AIDS-defining cancers, the SIR was 3.6 (95% CI: 2.9–4.3) among IDUs, 1.4 (95% CI: 1.1–1.8) among heterosexuals, and 2.0 (95% CI: 1.5–2.6) among MSM (Table 3).

Persons with HIV/AIDS who had less than 1-year interval between first HIV-positive test and AIDS diagnosis differed from other PWHA in many ways (Table 4). Among these late presenters, the contribution of person-years was much larger among heterosexuals, MSM, and PWHA born outside Italy, whereas HAART use was rarer and median CD4+ cell count at AIDS diagnosis was lower than in other PWHA. The SIR for KS (1252) was also higher in late presenters than in other PWHA (Table 4). Conversely, SIR for non-AIDS-defining cancers increased from 1.3 (95% CI: 1.0–1.7) in PWHA whose interval between first HIV-positive test and AIDS diagnosis was less than 1 year, to 2.8 (95% CI: 2.2–3.5) and 3.9 (95% CI: 2.9–5.0), respectively, in PWHA in whom the corresponding interval was 1–9 years and 10 years or more (Table 4).

## Discussion

Our study showed substantial changes in the cancer pattern of Italian PWHA after the introduction of HAART in 1996. Non-Hodgkin lymphoma replaced KS as the most frequent cancer type and non-AIDS-defining cancers increased from 15 to 25% of all cancers. For the first time a significant excess of liver cancer emerged in the Italian AIDS linkage study (Dal Maso *et al*, 2003), in agreement with record-linkage studies from the United States (Engels *et al*, 2006; Patel *et al*, 2008) and findings from HIV cohorts in Italy (Serraino *et al*, 2007) and Switzerland (Clifford *et al*, 2005). As PWHA live longer, the appearance of an excess of liver cancer compared with the general population was predictable owing to the high prevalence of hepatitis B and, more notably, hepatitis C infection among PWHA. An association between liver cancer risk and low CD4+ cell count in the year preceding liver cancer has recently been reported (Clifford *et al*, 2008), suggesting that immunodeficiency may contribute to the liver cancer excess in PWHA (Weber *et al*, 2006).

The risk of HL, myeloma, and cancers of the cervix, anogenital tract, lung, brain, and skin (non-melanoma) continued to be significantly increased among PWHA after 1996. The greatest cancer excess was found in proximity to AIDS diagnosis, but persisted in the 10 years afterwards. Notably, elevated SIRs were seen overall and in each HIV transmission category for cancers of the cervix and the anogenital tract that are, in the vast majority, associated with HPV infection (IARC, 2007). Hence, it is not yet clear whether the partial immune reconstitution induced by HAART will ultimately also have a favourable effect on HPV-associated cancers (Frisch *et al*, 2000; Dorrucci *et al*, 2001; Ahdieh-Grant *et al*, 2004; Heard *et al*, 2004). Inadequate coverage by cervical cancer-screening programmes of women living with HIV, despite ubiquitous access to HAART and regular contact with medical services, has been suggested as the main reason for the greater excess risk of ICC in countries such as Italy (Franceschi *et al*, 2006) and Spain (Galceran *et al*, 2007) compared with the United States and Northern Europe (Franceschi and Jaffe, 2007). According to a survey of HIV clinics in Italy (Murri *et al*, 2006), HIV care providers in Italy are well aware of screening needs, but they fail to achieve good coverage among HIV-positive women mainly for organisational reasons.

With respect to HL, our findings confirm previous reports (Dal Maso *et al*, 2003; Biggar *et al*, 2006; Engels *et al*, 2006; Patel *et al*, 2008), but, contrary to what has been suggested in the United States (Biggar *et al*, 2006), the SIR for HL did not increase compared with the pre-HAART period. It is noteworthy that MSM showed particularly elevated SIRs for HL though not for NHL in our study. The disappearance of excess risk for leukaemias in recent years suggests an improvement in the distinction between NHL and other lymphoid neoplasms (Dal Maso and Franceschi, 2003), but an elevated SIR for myeloma was confirmed in 1997–2004.

An increased risk for lung cancer among Italian PWHA was also confirmed (Grulich *et al*, 2007), but it is likely to derive mainly from the high proportion of smokers, notably among IDUs (Clifford *et al*, 2005). Conversely, we found no excess for head and neck cancers, which are also associated with smoking and, in a fraction of cases, HPV infection (Clifford *et al*, 2005; Kreimer *et al*, 2005). In respect to brain cancer, microscopic confirmation continues to be very rare and misclassification with other HIV-related diseases located in the brain cannot be ruled out.

Skin cancer (non-melanoma) was increased by two-fold in PWHA as in previous reports (Franceschi *et al*, 1998; Allardice *et al*, 2003; Dal Maso *et al*, 2003; Clifford *et al*, 2005). The excess risk observed in PWHA was confirmed, however, to be weaker than among transplant recipients (Grulich *et al*, 2007; Serraino *et al*, 2007).

Standardised incidence ratios for a broad range of cancer sites, including common neoplasms such as stomach, colon, breast, and prostate, were close to unity and hence compatible with no influence of immune status on the risk of several types of cancer.

Our present study has strengths and weaknesses. Strengths include the large number of AIDS cases and person-years available before and after HAART introduction. The completeness and quality of the AIDS Registry (Conti *et al*, 1997) and Italian CRs (Curado *et al*, 2007) have been shown to be satisfactory, and the linkage procedures are accurate (Dal Maso *et al*, 2001; Clifford *et al*, 2005). The limited population mobility, the strict rules for maintenance of ‘legal residence’ in Italy, and the possibility of verifing the vital status of PWHA with national mortality records provided reassurance on the accuracy of follow-up and allowed us to extend our observation period to 10 years after AIDS diagnosis. Censoring at 5 years after AIDS diagnosis would not, however, have modified our findings. Finally, microscopic or instrumental confirmation was available for most cancer sites for which we report risk increases. In particular, we were confident that no *in situ* carcinomas were misclassified as ICC or anal cancer.

Systematic reporting of HIV cases in Italy is limited to a few areas (Centro Operativo AIDS, 2008), and therefore a major weakness of our present study is reliance on AIDS case reporting only. The yearly number of AIDS cases has diminished three-fold in Italy between the peak in the mid-1990s and 2000 (Centro Operativo AIDS, 2008) and, most important, the meaning of AIDS onset has changed. Formerly an irreversible stage of HIV progression, AIDS often indicates, in the post-HAART era, poor adherence to treatment or development of resistance (Kaldor *et al*, 2009).

The availability (as from 1996) of information on the date of first HIV-positive test in the AIDS Registry allowed us, however, to focus on PWHA who had concomitant, or nearly concomitant, HIV infection and AIDS-defining illness. Such late presenters increased in Italy from 20.5% in 1996 to 55.5% of AIDS cases in 2007 (Centro Operativo AIDS, 2008). They were in the vast majority individuals who had acquired HIV through sexual intercourse and, unlike IDUs in the early phase of the HIV epidemic, did not perceive themselves as at high risk for the infection (Borghi *et al*, 2008). Persons with HIV/AIDS born outside Italy were also frequent. In addition, late presenters had never taken HAART and were severely immunocompromised more often than AIDS cases who had been HIV-positive for many years prior. With respect to cancer pattern, KS greatly predominated over all other tumours.

Our study showed that to prevent cancer in PWHA with increasing life expectancy, the use of HAART must be accompanied by more effective cancer-prevention strategies (Massad *et al*, 2004), notably antismoking, cervical cancer screening programmes, and, possibly, hepatitis C virus treatment. Improvements in the timely identification of HIV-positive individuals is also a priority in Italy to avoid the immunological deterioration associated with delayed HAART use, and also to provide a better tool to monitor the HIV epidemic (Borghi *et al*, 2008).

## Figures and Tables

**Table 1:** Cancer registry characteristics, AIDS diagnoses, and linked cancers from 24 Italian cancer registries

	**Reporting period**				**Linked cancers^c^**					
									**All cases**	
**Cancer registry**	**Pre-HAART**	**Post-HAART**	**Population ( × 1000)^a^**	**AIDS cases^b^**	**KS**	**NHL**	**ICC**	**Other**	**Pre-HAART**	**Post-HAART**
Alto Adige/Südtirol	1995–1996	1997–2002	460	230	7	4	0	4	7	8
Biella	1995–1996	1997–2002	189	268	4	8	1	3	6	10
Brescia	—	1999–2001	1012	1909	13	17	2	12	0	44
Ferrara	1991–1996	1997–2002	314	959	19	21	1	9	25	25
Florence	1985–1996	1997–2003	1162	1199	133	86	2	44	186	79
Friuli Venezia Giulia	1995–1996	1997–2003	1188	407	13	10	1	5	8	21
Genoa	1986–1996	1997–2003	920	1775	91	85	10	46	155	77
Macerata	1991–1996	1997–2000	293	135	7	3	1	1	6	6
Milan	—	1999–2002	1256	4822	28	40	3	34	0	105
Modena	1988–1996	1997–2004	615	633	45	36	2	11	48	46
Naples	1996	1997–2003	541	110	5	2	0	1	1	7
Parma	1978–1996	1997–2003	394	368	24	21	1	11	31	26
Ragusa	1981–1996	1997–2003	291	63	2	5	0	0	5	2
Reggio Emilia	1996	1997–2004	450	412	15	24	2	8	11	38
Romagna	1985–1996	1997–2004	803	1948	94	104	3	55	149	107
Salerno	1996	1997–2001	1088	223	4	4	1	2	0	11
Sassari	1992–1996	1997–2003	469	374	12	20	2	7	24	17
Sondrio	—	1998–2002	177	118	1	1	0	1	0	3
Syracuse	—	1999–2002	396	157	1	4	0	2	0	7
Trento	1995–1996	1997–2002	460	347	7	16	0	6	7	22
Turin	1985–1996	1997–2002	1091	1794	108	61	1	40	145	65
Umbria	1994–1996	1997–2003	831	435	18	28	1	9	30	26
Varese	1976–1996	1997–2002	800	1668	62	98	3	30	125	68
Veneto	1987–1996	1997–2002	2077	1599	88	74	2	42	129	77
										
Total			17 277	21 951	801	772	39	383	1098	897

HAART=highly active antiretroviral therapy, KS=Kaposi sarcoma, NHL=non-Hodgkin lymphoma, ICC=invasive cervical cancer.

a Observed population in 1997–2002.

b AIDS cases notified in cancer registry areas in 1986–2005.

c Cancers reported to cancer registries in people with AIDS, aged 16–69 years, between 1986 and 2004 from 5 years prior to 10 years after AIDS diagnosis (at/after AIDS for AIDS-defining cancers).

**Table 2:** Observed (Obs) and expected (Exp) cancers in persons with HIV/AIDS^a^, standardised incidence ratio (SIR), and corresponding 95% confidence interval (CI) by year of cancer diagnosis. Italy, 1986–2004

	**Year of cancer diagnosis**					
	**1986–1996 (56 643 py)**			**1997–2004 (45 026 py)**		
**ICD10; Cancer type or site**	**Obs**	**Exp**	**SIR (95% CI)**	**Obs**	**Exp**	**SIR (95% CI)**
*AIDS-defining cancers*						
C46; Kaposi sarcoma	507	0.3	1792 (1640–1956)	294	0.5	572 (508–641)
C82–C88, C96; NHL	420	0.8	497 (450–546)	352	3.8	93.4 (83.9–104)
C53; Cervix uteri	9	0.2	51.0 (23.1–97.3)	30	0.7	41.5 (28.0–59.3)
						
*Non-AIDS-defining cancers*						
C00–C14, C30–C32; Head and neck	6	4.4	1.4 (0.5–3.0)	11	6.0	1.8 (0.9–3.3)
C15; Oesophagus	0	0.6	—	2	0.8	2.5 (0.2–9.1)
C16; Stomach	6	3.2	1.9 (0.7–4.1)	6	3.9	1.6 (0.6–3.4)
C18; Colon	2	3.9	0.5 (0.0–1.9)	9	6.2	1.4 (0.7–2.7)
C19–C20; Rectum and rectosigmoid junction	5	2.0	2.5 (0.8–5.9)	7	3.1	2.3 (0.9–4.7)
C21; Anus	6	0.2	35.5 (12.8–77.7)	11	0.3	44.0 (21.8–78.9)
C22; Liver	3	1.4	2.1 (0.4–6.4)	16	2.5	6.4 (3.7–10.5)
C23–C24; Biliary tract	0	0.4	—	2	0.5	3.9 (0.4–14.5)
C25; Pancreas	2	1.2	1.7 (0.2–6.3)	2	1.8	1.1 (0.1–4.1)
C33–C34; Trachea and lung	17	8.2	2.1 (1.2–3.3)	42	10.3	4.1 (2.9–5.5)
C37–C38; Thymus, heart, mediastinum, pleura	1	0.3	3.9 (0.0–22.6)	1	0.3	3.3 (0.0–18.7)
C40–C41; Bone and articular cartilages	1	0.4	2.5 (0.0–14.0)	1	0.4	2.6 (0.0–14.6)
C43; Melanoma	3	3.4	0.9 (0.2–2.6)	3	5.3	0.6 (0.1–1.7)
C44; Skin non-melanoma	18	8.7	2.1 (1.2–3.3)	28	15.6	1.8 (1.2–2.6)
C45; Mesothelioma	0	0.3	—	1	0.4	2.2 (0.0–12.8)
C47, C49; Peripheral nerves, soft/connective tissues	0	0.8	—	3	0.9	3.2 (0.6–9.5)
C50; Breast^b^	3	4.0	0.8 (0.1–2.2)	5	8.7	0.6 (0.2–1.4)
C54; Endometrium	0	0.4	—	1	0.7	1.5 (0.0–8.3)
C56; Ovary	1	0.6	1.7 (0.0–9.7)	0	0.8	—
C51, C52, C57; Vulva and vagina	2	0.1	24.6 (2.3–90.6)	3	0.1	24.3 (4.6–71.8)
C55; Utero, unspecified	1	0.0	25.2 (0.0–145)	0	0.0	—
C60, C63; Penis	0	0.1	—	3	0.2	12.0 (2.3–35.5)
C61; Prostate	2	1.5	1.3 (0.1–4.7)	0	5.3	—
C62; Testis	5	3.5	1.4 (0.5–3.4)	2	2.9	0.7 (0.1–2.5)
C64–C66, C68; Kidney	3	2.5	1.2 (0.2–3.6)	3	4.0	0.7 (0.1–2.2)
C67; Bladder	3	4.5	0.7 (0.1–2.0)	2	6.4	0.3 (0.0–1.2)
C70–C72; Brain and central nervous system	8	2.3	3.5 (1.5–7.0)	8	2.5	3.2 (1.4–6.3)
C73; Thyroid	0	2.2	—	0	3.6	—
C81; Hodgkin lymphoma	47	2.6	18.0 (13.2–23.9)	37	1.8	20.7 (14.6–28.5)
C90; Multiple myeloma/plasma cell neoplasm	3	0.5	5.5 (1.0–16.4)	4	1.0	3.9 (1.0–10.0)
C91–C95; Leukaemias, all	11	2.2	4.9 (2.4–8.8)	3	2.7	1.1 (0.2–3.3)
C26, C39, C48, C76, C80; Unk/ill-defined primary site	3	1.2	2.5 (0.5–7.4)	5	1.3	3.9 (1.2–9.2)
						
Total non-AIDS-defining cancers	162	68.3	2.4 (2.0–2.8)	221	100.7	2.2 (1.9–2.5)

py=person-years, NHL=non-Hodgkin lymphoma, Unk=unknown.

a Cancers reported to cancer registries in people with AIDS, aged 16–69 years, between 1986 and 2004 from 5 years prior to 10 years after AIDS diagnosis (at/after AIDS for AIDS-defining cancers).

b Women only.

**Table 3:** Observed (Obs) cancers in persons with HIV/AIDS^a^, standardised incidence ratio (SIR), and corresponding 95% confidence interval (CI) by age group and HIV transmission category. Italy, 1997–2004

	**Gender**				**Age group**				**HIV transmission category**					
	**Men (*N*=17 173) (33 964 py)**		**Women (*N*=4891) (11 062 py)**		**16–34 (18 815 py)**		**35–69 (26 211 py)**		**IDU (21 715 py)**		**Heterosexual (15 021 py)**		**MSM (8290 py)**	
**ICD10; Cancer type or site**	**Obs**	**SIR (95% CI)**	**Obs**	**SIR (95% CI)**	**Obs**	**SIR (95% CI)**	**Obs**	**SIR (95% CI)**	**Obs**	**SIR (95% CI)**	**Obs**	**SIR (95% CI)**	**Obs**	**SIR (95% CI)**
*AIDS-defining cancers*														
C46; Kaposi sarcoma	271	550 (487–620)	23	1066 (675–1601)	88	386 (309–475)	206	720 (625–826)	59	204 (155–263)	91	777 (625–954)	144	1338 (1128–1575)
C82–C88, C96; NHL	275	85.6 (75.8–96.4)	77	138 (109–173)	100	78.9 (64.2–96.0)	252	101 (88.7–114)	152	87.3 (73.9–102)	126	106 (88.3–126)	74	88.2 (69.3–111)
C53; Cervix uteri	—	—	30	41.5 (28.0–59.3)	15	42.3 (23.6–69.9)	15	40.7 (22.7–67.3)	17	44.2 (25.7–70.9)	13	38.4 (20.4–65.9)	—	—
														
*Non-AIDS-defining cancers*														
C21; Anus	8	43.3 (18.5–85.7)	3	45.8 (8.6–136)	4	96.6 (25.1–250)	7	33.5 (13.3–69.4)	7	85.1 (33.7–176)	1	9.6 (0.0–55.1)	3	47.0 (8.9–139)
C22; Liver	14	5.9 (3.2–9.8)	2	20.3 (1.9–74.8)	4	24.3 (6.3–62.9)	12	5.2 (2.7–9.0)	11	22.2 (11.0–39.9)	2	1.8 (0.2–6.5)	3	3.5 (0.7–10.3)
C33–C34; Lung	38	3.9 (2.8–5.4)	4	6.4 (1.7–16.5)	8	18.3 (7.8–36.2)	34	3.5 (2.4–4.8)	19	11.0 (6.6–17.3)	11	2.2 (1.1–4.0)	12	3.3 (1.7–5.8)
C44; Skin non-melanoma	21	1.6 (1.0–2.5)	7	2.4 (1.0–5.0)	6	2.4 (0.9–5.2)	22	1.7 (1.0–2.5)	14	2.9 (1.6–4.9)	8	1.2 (0.5–2.3)	6	1.5 (0.5–3.2)
C81; Hodgkin lymphoma	35	25.9 (18.0–36.0)	2	4.6 (0.4–16.9)	11	13.2 (6.6–23.8)	26	27.2 (17.7–39.8)	16	18.4 (10.5–29.9)	8	13.8 (5.9–27.3)	13	38.6 (20.4–66.1)
														
Total non-AIDS-defining cancers	182	2.3 (2.0–2.7)	39	1.7 (1.2–2.4)	55	3.4 (2.6–4.4)	166	2.0 (1.7–2.3)	106	3.6 (2.9–4.3)	64	1.4 (1.1–1.8)	51	2.0 (1.5–2.6)

py=person-years, IDU=injecting drug users, MSM=men who have sex with men, NHL=non-Hodgkin lymphoma.

a Cancers reported to cancer registries in people with AIDS, aged 16–69 years from 5 years prior to 10 years after AIDS diagnosis (at/after AIDS for AIDS-defining cancers).

**Table 4:** Distribution of selected characteristics at AIDS diagnosis, observed (Obs) cancers^a^, standardised incidence ratio (SIR), and corresponding 95% confidence interval (CI) by time elapsed since first HIV-positive test and AIDS. Italy, 1997–2004

	**Time between first HIV-positive test and AIDS diagnosis (years)^b^**					
**Characteristics**	**<1 (14 868 py)**		**1–9 (13 994 py)**		**⩾10 (9028 py)**	
*HIV transmission category*						
IDU	13%		53%		84%	
Heterosexual	59%		30%		10%	
MSM	28%		17%		6%	
			
PWHA born outside Italy	25%		12%		4%	
			
Median age (years) at AIDS diagnosis (IQR)	39 (33–49)		36 (32–41)		38 (35–41)	
			
Median CD4 (cells/ml) at AIDS diagnosis (IQR)	47 (17–110)		80 (26–193)		102 (38–215)	
			
PWHA using HAART at AIDS diagnosis	7%		61%		66%	
						
**ICD10; Cancer type or site**	**Obs**	**SIR (95% CI)**	**Obs**	**SIR (95% CI)**	**Obs**	**SIR (95% CI)**
C46; Kaposi sarcoma	162	1252 (1067–1461)	77	444 (350–555)	35	414 (288–576)
C82–C85, C88, C96; NHL	114	100 (82.5–120)	110	91.9 (75.6–111)	104	187 (153–227)
C53; Cervix uteri	4	27.7 (7.2–71.7)	9	32.1 (14.5–61.1)	14	111 (60.7–187)
						
Total non-AIDS-defining cancers	58	1.3 (1.0–1.7)	74	2.8 (2.2–3.5)	57	3.9 (2.9–5.0)

py=person-years, IDU=injecting drug users, MSM=men who have sex with men, IQR=interquartile range (25–75 percentile), NHL=non-Hodgkin lymphoma.

a Cancers reported to cancer registries in people with AIDS, aged 16–69 years from 5 years prior to 10 years after AIDS diagnosis (at/after AIDS for AIDS-defining cancers).

b Twelve (7%) cancers and 19% of py were excluded, as date of first HIV-positive test was missing.

## Appendix

Members of the CARL study include: Antonella Zucchetto, Angela De Paoli (Epidemiology and Biostatistics Unit, Aviano); Americo Colamartini (Romagna Cancer Registry, Meldola); Mariangela Autelitano (Cancer Registry of Milan); Emanuele Crocetti (Tuscany Cancer Registry); Enza Marani (Genoa Province Cancer Registry, Genova); Anna Rita Fiore (Registro Tumori del Veneto, Padova); Andrea Tittarelli (Registro Tumori Lombardia – Provincia di Varese); Stefano Rosso (Piedmont Cancer Registry, City of Torino); Ivan Rashid (Modena Cancer registry); Francesco Donato (Brescia Health Unit Cancer Registry); Annamaria Pezzarossi (Reggio Emilia Cancer Registry); Paolo Sgargi (Parma Province Cancer Registry); Francesco La Rosa (Umbria Cancer Registry); Silva Franchini (Trento Cancer Registry); Loris Zanier (Friuli Venezia Giulia Cancer Registry); Rosaria Cesaraccio (Cancer Registry of Sassari); Gennaro Senatore (Salerno Cancer Registry); Pier Carlo Vercellino (Piedmont Cancer Registry, Province of Biella); Fabio Vittadello (Alto Adige/Südtirol Cancer Registry); Raffaele Palombino (Campania Cancer Registry); Maria Lia Contrino (Syracuse Province Registry of Pathology); Silvia Antonini (Macerata Cancer Registry); Sergio Maspero (Sondrio Cancer Registry); Maria Guglielmina La Rosa (Ragusa Cancer Registry); Stefano Boros, Maria Cristina Salfa (National Institute of Health, Rome)
